# Effect of radiation exposure on oral mucosal cells: An *in vitro* study

**DOI:** 10.6026/973206300210823

**Published:** 2025-04-30

**Authors:** Silpi Chatterjee, Pallavi Choudhary, Asim Mustafa Khan, Reshma V.J, Muhaseena Muhamood, Arpita Maitra

**Affiliations:** 1 Department of Public Health Dentistry, Dr. D.Y.Patil Dental College & Hospital, Dr D.Y.Patil Vidyapeeth, Pune, Maharashtra, India; 2 Department of Oral and Maxillofacial Surgery, Consultant at Ramakrishna mission hospital and T.B sanatorium, Consultant at K.C Roy memorial hospital and heart centre, Ranchi, Jharkhand, India; 3 Department of Biomedical dental sciences, College of dentistry Imam Abdulrahman Bin Faisal University, Dammam, Saudi Arabia; 4 Department of Oral Medicine and Radiology, Guru Nanak Institute of Dental Sciences and Research, Kolkata, West Bengal, India

**Keywords:** Radiation exposure, oral mucosal cells, *in vitro* study, cytotoxicity, genotoxicity, DNA damage, cell viability, radiation-induced damage

## Abstract

Radiation generates toxic reactions and DNA damage in oral mucosal cells which depends on the exposure dose during laboratory tests. The survival rate of cells dropped from 98.6% in non-irradiated controls to 40.8% at 8 Gy while both structural cell alterations and DNA damage manifestations became evident. The extent of DNA damage in cells increased as radiation doses became higher according to findings from the Comet assay. Radiation produces marked impact on the defense capacity of oral tissues. Protective measures must be implemented as an essential way to prevent damage to oral mucosa tissues exposed to radiation.

## Background:

Radiation exposure has become a significant concern due to its extensive use in medical imaging, radiotherapy and various industrial processes. While radiation plays a pivotal role in diagnostic and therapeutic procedures, it poses potential risks to normal tissues, particularly rapidly dividing cells, including those of the oral mucosa [1]. Oral mucosal cells are especially vulnerable to radiation-induced damage, which may manifest as cytotoxicity, genotoxicity, inflammation and impaired cellular functions [2]. Epithelial cell cultures exhibited superior expression of stem cell, proliferative, Notch signalling and autophagy markers compared to oral and conjunctival cells, indicating their stronger regenerative potential [3]. Low pulse energy, high repetition rate Nd:YAG laser irradiation enhances cell proliferation and differentiation in oral microenvironment cells, especially osteoblasts, without compromising cell viability [4, 5]. The majority of p63 and Ki-67 immuno-positive cells were located in the basal layer whereas cytoplasmic involucrin expression was seen in the supra-basal layers, similar to native canine buccal mucosa [6]. Unlike histology, scanning acoustic microscopic measurements are non-invasive and can be used to monitor tissue graft development without damaging any cells/tissues [7]. Conventional radiotherapy for head-and-neck tumors induces a rapid decline in oral mucosal cell proliferation and density, particularly during the first week of treatment [8]. Topical application of Tat-Smad7 significantly promotes healing of radiotherapy-induced oral mucositis by reducing inflammation, DNA damage and apoptosis in mucosal cells while enhancing keratinocyte proliferation [9, 10]. However, the effects of varying radiation doses on oral mucosal cells remain inadequately explored, particularly with regard to cellular viability, morphological changes and DNA integrity. Therefore, it is of interest to evaluate the impact of different doses of radiation on oral mucosal cells using a comprehensive *in vitro* approach.

## Materials and Methods:

## Study design and cell culture:

This *in vitro* study was conducted using oral mucosal cells obtained from healthy volunteers (n=10) aged between 18-35 years. Written informed consent was obtained from all participants before sample collection. The cells were collected through exfoliative cytology using sterile wooden spatulas and were cultured in Dulbecco's Modified Eagle Medium (DMEM) supplemented with 10% fetal bovine serum (FBS), 1% penicillin-streptomycin and 1% L-glutamine. The cells were incubated at 37°C in a humidified atmosphere with 5% CO2 until reaching approximately 80% confluency.

## Experimental groups and radiation exposure:

The cultured cells were divided into four groups based on the radiation dose received:

[1] Group I (Control): No radiation exposure.

[2] Group II (Low Dose): Exposed to 2 Gy of radiation.

[3] Group III (Medium Dose): Exposed to 5 Gy of radiation.

[4] Group IV (High Dose): Exposed to 8 Gy of radiation.

Radiation exposure was administered using a linear accelerator (LINAC) with photon energy of 6 MV. Cells were exposed to radiation at a dose rate of 2 Gy/min.

## Cell viability assay (MTT assay):

Cell viability was assessed using the MTT assay, which measures mitochondrial activity as an indicator of cellular metabolic activity. Following radiation exposure, 100 µL of MTT solution (0.5 mg/mL) was added to each well and incubated for 4 hours at 37°C. The resulting formazan crystals were dissolved in 200 µL of dimethyl sulfoxide (DMSO) and absorbance was measured at 570 nm using a microplate reader. Viability was expressed as a percentage of the control group.

## Morphological assessment:

Cellular morphology was evaluated using phase-contrast microscopy. Cells were visually examined for structural changes, including cell shrinkage, membrane blebbing and nuclear condensation, which are indicative of apoptosis or necrosis. Representative images were captured and analyzed.

## DNA damage analysis (Comet assay):

The comet assay (single-cell gel electrophoresis) was performed to evaluate DNA damage in individual cells. Following radiation exposure, cells were embedded in low-melting-point agarose on microscope slides and subjected to electrophoresis under alkaline conditions (pH > 13). After staining with ethidium bromide, DNA migration patterns were visualized using a fluorescence microscope. Tail length, tail intensity and tail moment were measured to assess the extent of DNA damage.

## Statistical analysis:

Data were analyzed using one-way ANOVA followed by post hoc Tukey's test to determine significant differences between groups. Results were expressed as mean ± standard deviation (SD), with a significance level of p < 0.05.

## Results:

The cell viability results obtained from the MTT assay demonstrated a significant decrease in viability with increasing radiation doses. The mean viability percentages for each group are presented in Table 1. Group I (Control) exhibited the highest viability, while Group IV (High Dose) showed the most substantial reduction in cell viability. Statistical analysis revealed significant differences between the control group and all irradiated groups (p < 0.05). The data indicate a clear dose-dependent decrease in cell viability, with Group IV showing a viability reduction of approximately 58% compared to the control group (Table 1). Microscopic examination of the cells revealed noticeable morphological alterations in response to radiation exposure. The control group displayed healthy, adherent cells with regular cell membranes and uniform cytoplasmic structures. In contrast, cells exposed to radiation (Groups II, III and IV) exhibited features suggestive of apoptosis and necrosis, including cell shrinkage, membrane blebbing and nuclear condensation. The severity of these changes increased with the radiation dose, with Group IV exhibiting the most profound alterations. The comet assay demonstrated a significant increase in DNA damage with escalating radiation doses. The parameters used to evaluate DNA damage included tail length, tail intensity and tail moment. The results are summarized in Table 2. The results demonstrated a significant increase in all DNA damage parameters (tail length, tail intensity and tail moment) in Groups II, III and IV compared to the control group (Table 2). The highest values were observed in Group IV, indicating severe DNA fragmentation and damage.

## Discussion:

The present *in vitro* study investigated the effects of varying radiation doses on oral mucosal cells, focusing on cell viability, morphological changes and DNA damage. The findings indicate a clear dose-dependent relationship between radiation exposure and cellular damage, which aligns with previous research on radiation-induced cytotoxicity and genotoxicity [1, 2]. Preferential use of limbal-derive cells in ocular surface reconstruction, providing key molecular insights into transplantation outcomes in corneal repair [3, 4]. Radiation exposure is known to induce oxidative stress through the generation of reactive oxygen species (ROS), leading to cellular apoptosis and necrosis [5]. Furthermore, the loss of cell viability noted in the high-dose group (8 Gy) suggests that oral mucosal cells are particularly sensitive to higher radiation doses, highlighting the need for protective strategies during clinical radiation applications [6]. Morphological alterations, including cell shrinkage, membrane blebbing and nuclear condensation, were evident in irradiated cells. These changes are hallmarks of apoptosis, which occurs following radiation-induced DNA damage and oxidative stress [7]. Studies have reported that high radiation doses can compromise cellular architecture, leading to loss of membrane integrity and disruption of cellular homeostasis [8, 9]. Tat-Smad7 does not impair the therapeutic efficacy of radiotherapy on adjacent oral cancer cells, instead increasing oxidative stress and apoptosis, supporting its safe use in clinical oncology settings [10]. Increased tail length, tail intensity and tail moment, as observed in this study, indicate extensive DNA fragmentation, which is consistent with the findings of previous studies evaluating radiation-induced DNA damage in oral cells [11, 12]. Interestingly, the findings from the comet assay are comparable to previous reports where oral keratinocytes and fibroblasts exhibited DNA fragmentation following exposure to therapeutic radiation doses [13]. The dose-dependent increase in DNA damage parameters further supports the notion that higher radiation doses are associated with greater genotoxicity [14]. Several studies have highlighted the importance of assessing radiation-induced cellular damage in oral mucosal cells, especially in patients undergoing radiotherapy for head and neck cancers [15]. Measures to reduce radiation-induced toxicity, such as radioprotective agents and optimized radiation protocols, have been proposed to minimize adverse effects [6]. Furthermore, the potential cumulative effects of repeated diagnostic imaging procedures involving ionizing radiation, such as X-rays and CT scans, should not be overlooked. Frequent exposure to low-dose radiation can result in sub-lethal damage that may accumulate over time, contributing to long-term adverse effects [7]. The present study has certain limitations, including the *in vitro* nature of the experiment, which may not entirely replicate the in vivo environment. Additionally, the study focused solely on oral mucosal cells, whereas other cellular components of the oral cavity, such as fibroblasts and endothelial cells, may also be affected by radiation exposure [8]. Future studies should consider a broader spectrum of oral tissues and employ advanced techniques for assessing radiation-induced damage.

## Conclusion:

Dose-dependent relationship underscores the importance of adopting protective measures during radiation therapy and diagnostic imaging. However, further studies are needed to explore potential protective agents that could mitigate radiation-induced damage in oral tissues.

## Figures and Tables

**Table 1:** Cell viability percentage in different radiation groups (Mean ± SD)

**Group**	**Radiation Dose (Gy)**	**Cell Viability (%)**
Group I	0 (Control)	98.6 ± 1.2
Group II	2	87.4 ± 2.3
Group III	5	65.2 ± 3.0
Group IV	8	40.8 ± 2.8

**Table 2:** DNA damage parameters in different radiation groups (Mean ± SD)

**Group**	**Tail Length (µm)**	**Tail Intensity (%)**	**Tail Moment (µm)**
Group I	2.1 ± 0.4	1.8 ± 0.5	0.45 ± 0.08
Group II	5.8 ± 1.1	6.5 ± 1.2	1.56 ± 0.22
Group III	12.3 ± 1.9	15.4 ± 2.1	3.48 ± 0.38
Group IV	20.6 ± 2.4	28.7 ± 2.8	6.95 ± 0.72
